# Supplementary material for: MetaRibo-Seq measures translation in microbiomes
Source: Nat Commun. 2020 Jun 29;11:3268. doi: 10.1038/s41467-020-17081-z (PMC7324362; doi:10.1038/s41467-020-17081-z)
Supplement: Supplementary file 10 — Supplementary Data 7 [file 41467_2020_17081_MOESM10_ESM.zip › File2/Confidence_High_Taxonomy/53302_out.krona.html]

Javascript must be enabled to view this page.

members
magnitude
magnitudeUnassigned
count
unassigned
taxon
rank

53302\_out

2

1
2
superkingdom

1
phylum
1239

1
class
186801

186802
order
1

1
family
31979

1
1485

SRS147557\_contig\_number\_contig-100\_24611.71749
genus

1

SRS147557\_contig\_number\_contig-100\_10444.10445
